# Does health-related quality of life predict injury event?

**DOI:** 10.5249/jivr.v1i1.9

**Published:** 2009-07

**Authors:** Hamid Soori, Kambiz Abachizadeh

**Affiliations:** ^*a*^Shahid Beheshti University of Medical Sciences- Tehran-Iran.

## Abstract

**Background:**

Unintentional injury is a leading threat to children's health. Some human factors have been determined as predictor of unintentional injury. Association between Health-Related Quality of Life (HRQOL) as a human factor and unintentional injuries is unclear. The objective of study is to examine the association between HRQOL and unintentional injuries among primary school children.

**Methods:**

This study was a cross-sectional conducted in Ahwaz, a city in Iran. Overall, 3375 children aged 6-10 years were randomly selected from primary school. HRQOL was measured by 56 items taken from seven domains of Netherlands Organization for Applied Scientific Research Academic Medical Center (TNO AZL) child quality of life (TACQOL) parent form. Parents were interviewed to collect information about incidence, cause and a brief description of injury within the past 12 months prior to the study.

**Results:**

The response rate was 3375 of 3792 (89%). There was a significant trend for increasing occurrence of injury with decreasing of HRQOL score (p was less than 0.001). Adjusted OR for injury was significantly higher in very low (2.38, 95% CI: 1.45-3.86), low (2.18, 95% CI: 1.34-3.56), and medium (1.73, 95%CI: 1.06-2.83) HRQOL groups compared to reference group (very high HRQOL). The median of total HRQOL (P less than 0.001) and all its domains (P=0.017) (except autonomous functioning) was lower in injured group compared to uninjured one.

**Conclusions:**

This study found an association between HRQOL and unintentional injury among primary school children. This is a preliminary finding and further investigations with a well-defined analytical design are needed.

## Introduction

Unintentional injury is a leading threat to children's health.^1,2,3^ It is estimated that in some European countries one in four children receives medical attention for an injury each year, in either primary or secondary care.^4^ According to national report of burden of diseases in Iran, at the age of 5 to 14, 59% of burden of diseases in males, 48% in females and 54% in both sexes is attributable to injuries.^5^The high proportion of children and young adults and the substantial socio-economic consequences of childhood injuries in less developed countries require prudent attention to the issue of injury control.^6^ The identification of the characteristics that contribute to injury risk is critical to the development and evaluation of pediatric injury prevention strategies.^7^.

Injuries result from a predictable interaction among host, environment, and injury agents.^7^ The significant association of child behavioral characteristics with injury risk supports recommendations by previous researchers that child behavioral characteristics be considered as a potential predictor in childhood injury research.^8,9,10^There is increasing evidence that children differentially engage in risk behaviors based on individual-difference characteristics (e.g., behavioral intensity and inhibitory control).^11^

Health-related quality of life (HRQOL) has been recognized as an important health outcome, some contend the most important outcome in child health services research.^12,13^ Measuring HRQOL is performed by assessing different domains like physical, motor, autonomous, cognitive, social and emotional functioning.^14^ No other study, to our knowledge, has documented association between HRQOL as a personal factor and probability of occurring risk taking behavior or unintentional injury. However, there are some studies which have examined the association between unintentional injury and risk factors related to some domains of HRQOL. For example, association between self-reported poor health, poor ability to concentrate and emotion-based factors with injuries has been investigated.^15,16^Risk taking behavior has been related to aspects of temperament, aggressive behavior, perceptual analysis skills, beliefs about injury vulnerability and estimation of physical abilities.^17^These risk factors might be used as proxies for HRQOL domains. So, domains of HRQOL and consequently total HRQOL might be associated with occurrence of unintentional injury.

There are still some ambiguities in respect to how and in what ways HRQOL effects unintentional injury. This is despite the existing association between domains of HRQOL and injury events. The relationship between HRQOL and risk taking behavior or human errors might be other mechanisms of injury event. The aim of this study is to test if HRQOL is associated with unintentional injuries among children aged 6-10 years old. The primary hypothesis of the study is to test if the incidence of unintentional injuries will be higher among children with lower HRQOL score.

## Methods

From a total of 400 primary schools in Ahwaz, a city in Iran, with 128,499 pupils, 76 schools were randomly selected in five different educational districts according to the number of students. Schools were stratified by number of pupils in each one, and by private/state type. From each school 50 students aged 6 to 10 (10 students per classroom, between grades 1-5) were randomly selected. Parents (mothers) of selected pupils were invited to their children’s schools on different occasions by an invitation letter. Mothers (but not children) completed and signed an informed consent form. Children who were living without a mother or both parents were excluded (2% of children). For each school two trained interviewers completed the questionnaires.

The study questionnaire measured demographic characteristics of the students and their parents including: child’s gender, age, and birth order, as well as number of children in the family and mother’s education level. Outcome variable included frequency, type, and causes of unintentional injury events during the past 12 months. The definition of unintentional injury used in this study followed the International Classification of Diseases-version 10 (S00-T98 and V01-Y98).^19^ In other words, for an event to be accounted as an “injury” it must have caused the individual to be hospitalized and receive care. Therefore, all mild to somewhat moderated injuries were exclude from the study and only moderate to severe injuries were included.

HRQOL consisted of 56 items taken from seven domains of Netherlands Organization for Applied Scientific Research Academic Medical Center (TNO AZL) child quality of life (TACQOL) parent form.^18^ Seven domains of HRQOL were physical, motor, autonomous, cognitive and social functioning, positive and negative emotions. In each item, the frequency of occurrence of domains was assessed. All domains were computed by totaling “always,” “often,” “only within the past few weeks,” “occasionally,” and “never.” Items were scored by assigning a value of 5 for “always” to 1 for “never” for positive well-being items (e.g., social functioning) ,and 5 for “never” to 1 for “always” for negative well-being items (e.g., negative emotions). Total scales of HRQOL scored from 56 to 280 (8-40 for each domain), with higher score indicating better HRQOL. The reason for this type of categorization was clarifying association between HRQOL and unintentional injury and probability of calculating odds ratio. More details about the measuring of children’s HRQOL are available elsewhere.^19^ For this study, the English version of the questionnaire was translated in Persian language.

**Data Analysis**

Statistical package for social sciences (SPSS- version 11.5) was employed for all data analyses. Mann-Whitney test was employed to compare HRQOL and its seven domains in injured and uninjured children because the data of HRQOL were severely left skewed. Scale reliability for each domain of HRQOL was assessed using Cronbach alpha.

The association between HRQOL and occurrence of unintentional injury was assessed using binary logistic regression. Only significant variables from the unadjusted logistic regression models were used for adjusted logistic regression analysis to assess the association of each level of HRQOL and injury events. Child’s sex, age, birth order, number of children in family and mother’s education level were used as confounding variables.

Results are presented as cross-tabulations, unadjusted and adjusted odds ratio (OR) with 95% confidence interval (CI). Chi square trend test (linear by linear association) was employed if there was a trend for children with lower HRQOL to have a higher incidence of injury. There was little missing data in this study which did not have any effect on the results.

## Results

Children were grouped into five separate categories based on their total HRQOL scores: (56-225 [very low HRQOL], 226-245 [low], 246-259 [medium], 260-269 [high], and 270-280 [very high]. The Cronbach alpha was 0.74, 0.75, 0.73, 0.81, 0.89, 0.73, 0.90 and 0.77 for total HRQOL, body, motor, cognitive, autonomous, social functioning and positive and negative moods, respectively.

The response rate was 3375 of 3792 (89%). The average age of mothers and their children were 34.9 years (SD=6.4), and 7.9 years (SD=1.5), respectively. Overall, 96.7% of children were living with both parents, and 49.3% were males.

Of all parents who participated in the study, 210 reported their children’s injury event within the past 12 months prior to the date of study. The incidence rate (I.R) of unintentional injury was 6.2% (95%CI: 5.5-7.1%, n=3375). The top five causes of unintentional injuries were from falls (51.6%, I.R= 3.2%, n=108), transport accidents (25.1%, I.R=1.6%, n=53), struck by thrown, projected or falling object (11.2%, I.R=0.7%, n=24), poisoning (5.1%, I.R=,0.33%, n=11) and burns & scalds (4.7%, I.R=0.3% , n=10).

As was mentioned above, HRQOL score range from 56 to 280 with higher score indicating better HRQOL. The incidence rates of unintentional injury were 8.3%, 7.9%, 6.3%, 5.0% and 3.7% for very low, low, medium, high and very high groups of HRQOL, respectively. The linear-by-linear association indicated that there was a significant trend for increasing occurrence of injury with decreasing of HRQOL (P<0.001).

Among the demographic variables in the study only gender was statistically significant between injured and uninjured group (P<0.001). In other words, the incidence rate of unintentional injury among boys was higher compared to girls (8.1% versus 4.4%). We were not able to detect any other statistically significant association for children’s mean age, mother’s educational level, child’s birth order and number of children in family between injured and uninjured groups (Table 1). Unadjusted OR of injury event and adjusted OR by sex were calculated for each level of HRQOL. Odd Ratios was significantly higher in very low, low, medium HRQOL groups compared to reference group (very high HRQOL). There was no significant difference between very high and high HRQOL groups (Table 2).The median score for all domains of HRQOL were compared between injured and uninjured groups (Table 3). In all domains we detected lower median score among injured group compared to uninjured and the difference was statistically significant. (P<0.001) (Table 3).

**Table:** Table 1. **Number (%) and 95% confidence interval of injury event by child’s sex, mother’s educational level, child’s birth order and number of children in family.**

	Total number	Number (%) ofinjury event	95% CI	P value
Sex				<0.001
Male	1663	134 (8.1%)	(6.8-9.4)	
Female	1712	75 (4.4%)	(3.4-5.4)	
Mother’s educational level				0.339
Illiterate	556	26 (4.8%)	(3.0-6.6)	
Primary	975	71 (7.3%)	(5.7-8.9)	
Middle	610	34 (5.6%)	(3.8-7.4)	
High school or higher	1220	78 (6.4%)	(5.0-7.8)	
Child’s birth order				0.260
1	959	53 (5.5%)	(4.2-6.8)	
2 and 3	1278	90 (7.1%)	(5.7-8.5)	
>3	1131	64 (5.7%)	(4.3-7.1)	
Number of children in family				0.404
1 and 2	1042	66 (6.4%)	(4.9-7.9)	
3 and 4	1296	86 (6.7%)	(5.3-8.1)	
>4	1037	55 (5.4%)	(4.0-6.8)	

* Total numbers reported are less than total subject numbers (3375) and injury events (210) due to incomplete reporting

**Table:** Table 2. Association between levels of Health-Related Quality Of Life (HRQOL)* and occurrence of unintentional injuries** among children within the year prior to the date of data collection.

HRQOL**	Unadjusted OR	P Value	Adjusted OR***	P Value
(n=649) Very high	1.00		1.00	
High (n=661)	1.37(0.80-2.31)	0.247	1.40(0.82-2.36)	0.215
Medium (n=719)	1.73(1.06-2.83)	0.033	1.73(1.06-2.83)	0.033
Low (n=680)	2.22(1.35-3.60)	0.002	2.18(1.34-3.56)	0.002
Very low (n=661)	2.34(1.43-3.82)	0.001	2.38(1.45-3.86)	0.001

* HRQOL scores were 56-225 for very low, 226-245 for low, 246-259 for medium, 260-269 for high, and 270-280 for very high.

** The incidence rates of unintentional injury were 8.3%, 7.9%, 6.3%, 5.0% and 3.7% for very low, low, medium, high and very high groups of HRQOL, respectively.

*** Adjusted by sex.

**Table:** Table 3. **Comparison of HRQOL median and its domains among injured and uninjured children**

	Injured (n=210)	Uninjured (n=3165)	
	Median	IQ Range*	Median	IQ Range*	P Value
Body functioning	36	7.0	39	5.0	<0.001
Motor functioning	39	4.0	40	3.0	0.010
Autonomous functioning	40	4.0	39	5.0	0.131
Positive emotions	34	9.2	36	10.0	0.017
Negative emotions	33	8.0	35	7.0	<0.001
Cognitive functioning	36	8.0	38	7.0	0.007
Social functioning	37	6.0	38	5.0	0.005
Total HRQOL	245	35.2	254	34.0	<0.001

* IQ Range: Inter Quartile range

## Discussion

This is the first study, to our knowledge, aimed to assess association between HRQOL and occurrence of unintentional injury in a group of- school children age 6-10). We set to test the hypothesis that the incidence of unintentional injury will be higher among children with lower score on HRQOL. We were able to identify the incident rate of 6.2% for unintentional injury among this sample of school children. Furthermore, children with lower levels of HRQOL were more likely to report higher incident of intentional injury; over two times more for very low and low group, and nearly two times more for medium group. Our findings also revealed that children in injured group had lower median score on body functioning, motor functioning, positive emotion, negative emotion, cognitive functioning, and social functioning, as well as overall median score for HRQOL (P<0.05). However, this was not the case for the “autonomous functioning” domain”. Similar to other studies ^15,16,20,21,22^, males had higher injury rates than females and the most frequent cause of injury was fall. 

Existing literature on the impact of HRQOL and its domains on unintentional injury are limited. In one study authors reported self-reported poor health status (as a proxy measure for body functioning domain of HRQOL) to be a risk factor for incidence of injury among school children.^15^ Other findings suggest that cognitive factors could predict childhood injury.^1,8^ Cognitive factors can influence children’s decision making process in engaging or not engaging in risky behaviors.^1^ 

The judgment of risk has been reported to be related to self-reported risk taking behavior.^20,23^ Children who underestimate the environmental risk or overestimate their physical ability have a higher rate of unintentional injury.^20,21,23^ Also, difficulty in concentration or easy diversion of attention increases the risk of injury.^15^ These risk factors might be related to cognitive functioning domain of HRQOL.

There are other probable risk factors related to positive and negative emotions and social functioning domains of HRQOL. For example, child’s temperament is associated with the risk of unintentional injury.^17,21,22^ In addition, aggressiveness, oppositional, impulsive and under-controlled behavior predict an increase risk of subsequent and concurrent injury event.^8,17,20,23^ Antisocial behaviors, discipline problems, disruptive behaviors and psychological problems have been reported as other risk factors of injury.^8,11,24,25^ Children with a disabling condition from vision/hearing disability, ADHD (Attention Deficit Hyperactivity Disorder), or chronic asthma had a significantly higher risk for nonfatal injuries compared with children without a disabling condition.^26^

Few published studies have examined the role of motor ability in increasing the risk of unintentional injury among children. These studies have reported mixed results.^27^ In our study we found no statistically significant association between children’s autonomous functioning and risk of injury. Autonomous functioning was not different between injured and uninjured children in our study.

**Limitations**

There are several limitations to the findings of this study. The inherent weakness of cross-sectional studies is applicable to our results. These types of studies only provide association between variables and lack ability to identify casual relationship. So, longitudinal studies are needed to establish such relationships. In this study, the translated questionnaire to Persian language was validated; but, cultural differences with other places in Iran and other countries decrease the generalizability of this study. Although, some probable confounding variables were controlled but there are still other potential confounding variables that should be considered for similar studies in the future, including; parent’s surveillance, environmental agents and socioeconomic status. Other limitation of the study has to do with the problem of “recall bias”. Participating parents could have had difficulty remembering injury events and subsequent health care utilizations including hospitalization since the questionnaire require them to remember events over a period of 12 month.

Nevertheless, findings from this study fill in some of the gaps that exist in the HRQOL literature regarding the consequential role of this variable in unintentional injury events. Furthermore, in this study, children HRQOL was measured with TACQOL, a generic instrument^27^ , while researchers who have attempted to address the issue of quality of life and injury often have used a very narrow definition of this term.^19-28^ Also, the high response rate (89%) gives credibility to the results of our study. Moreover, this was a population-based study and the subjects were highly representative of the children in the city.

In conclusion, this study found an association between HRQOL and unintentional injury. This is a preliminary finding and further investigations with a well-defined analytical design need to be done. Further clarification of the role of HRQOL and its domains as potential predictors of unintentional injury not only can complement injury literature, but help to refine current injury prevention strategies and interventions to reduce prevalence of injury among children.

The best way to decrease incidence of unintentional injuryis prevention.^15,20,22^ Injury scholars have long debated the relative value of environmentally oriented injury prevention strategies versus person-oriented.^22^ This study suggests that preventive strategies, while addressing broader community and environmental risk factors for injuries, need to address the human factors such as quality of life associated with these injuries. Also, it improves the concept of HRQOL application as a predictor. HRQOL is usually used as outcome in research, seldom used as a predictor.^12,13^
